# BigMove: A Group Intervention for People with Physical and Mental Health Conditions: Development, Theoretical Frameworks, Essential Elements and Design

**DOI:** 10.5334/ijic.5955

**Published:** 2022-04-19

**Authors:** Sabina van der Veen, Natalie Evans, Marijn C. Aalders, Louis G. Overgoor, Martijn A. Huisman, Guy A. M. Widdershoven

**Affiliations:** 1 Department of Ethics, Law and Humanities, Amsterdam UMC, Vrije Universiteit Amsterdam, Amsterdam Public Health Institute, Amsterdam, The Netherlands; 2 Bettery Institute, Diemen, The Netherlands; 3 Department of Epidemiology and Data Science, Amsterdam UMC, Vrije Universiteit Amsterdam, Amsterdam Public Health Institute, Amsterdam, The Netherlands

**Keywords:** group intervention, comorbidity, Capability Approach, Self Determination Theory, mental health conditions, ICF

## Abstract

**Introduction:**

This article describes an innovative, integrated care intervention, called BigMove, which aims to improve the functioning, capabilities and quality of life of people with a combination of physical and mental health conditions.

**Description:**

Theoretical frameworks reflected in the intervention are the Capability Approach (CA) and Self-Determination Theory (SDT). Essential elements of the intervention included to expand participants’ behavioural repertoire are motivational interviewing; functional goal setting (using the International Classification of Functioning, Disability and Health (ICF); cognitive behavioural therapy; enjoyment; support of the group; and physical activity. The design combines individual sessions and group sessions.

**Discussion:**

By integrating the CA and the SDT, the intervention enables participants to make self-directed and value-driven choices in life and change their behaviour accordingly to strengthen their functioning and capabilities. To foster person-centred, integrated care, it is crucial to reform the interaction between professionals and patients and to re-structure the organisation and financing of care to enable the provision of complex integrated care interventions.

**Conclusion:**

For people with physical and mental health conditions, the intervention BigMove provides an innovative integrated care approach that addresses aspirations people have regarding their functioning and focuses on individual goal setting and behaviour change.

## Introduction

Because of the increasing prevalence of people living with more than one chronic health condition (multimorbidity) healthcare systems and policymakers worldwide face a growing public health challenge [1,2]. There is a strong relationship between chronic physical health conditions and mental health conditions: people with physical health conditions are more likely to suffer from depression. In older adults, having physical health conditions combined with depression or cognitive impairment is associated with a greater prospective disability than physical health conditions alone [3], and significantly poorer health outcomes and reduced quality of life [4]. The burden of physical and mental health conditions is not limited to older people; two thirds of people with a combination of mental and physical health conditions are under the age of 65 [1,5]. The relationship between physical and mental health conditions is assumed to be bi-directional [6].

People with a combination of physical and mental health conditions are frequent users of ambulatory and inpatient care [1]. Much of this care is not only expensive but also ineffective [7]. A review on the effectiveness of interventions in primary care and community settings for people with more than one chronic health problem showed mixed results and no clear improvements on “clinical outcomes, health service use, medicine adherence, patient-related health behaviours, health professional behaviours or cost” [8, p. 2]. A probable cause of the ineffectiveness of care is that the provision and financial reimbursement of health care is arranged to address the diagnosis and treatment of single diseases, according to the dominant biomedical model. These single, disease-oriented approaches are not effective, efficient, and increasingly inappropriate for the management of multiple chronic conditions [9,10]. This is even more relevant when patients suffer from a combination of physical and mental health conditions. A failure to treat physical and mental health in an integrated way results in fragmentation of care, poor communication between specialists, low satisfaction with care and poor care outcomes [11,12]. It is suggested that care for people with physical and mental health conditions is likely to improve if mental health specialists and other professionals collaborate closely in the delivery of care [4]. The WHO has acknowledged an urgent need for integrated care to manage mental disorders and other chronic diseases to “cater to the overall health needs of the person” [13, p. 42].

In this article, we describe the intervention BigMove. BigMove aims to improve participants’ functioning, based on participants’ personal goals, and to foster participants’ feeling of control over their functioning. The perspective and goals of the participant are leading, and professionals collaborate in the delivery of patient-centred care and integrated care which is needed for successful and sustainable care for people with physical and mental comorbidities [14]. The intervention corresponds with the definition of integrated care as formulated by National Voices in 2013; “I can plan my care with people who work together to understand me and my carer(s), allow me control, and bring together services to achieve the outcomes important to me.”

The aim of this article is to provide details about the intervention’s development, including the theoretical frameworks and the essential elements of the intervention. We describe the design of the intervention, provide a case example, and reflect on the intervention’s strengths and limitations of the intervention. The article follows guidelines for the reporting and replicability of interventions [15].

## Description of the care practice

### Development

The basic idea for the intervention BigMove was developed in 2003 by a general practitioner and a physiotherapist practising in an ethnically diverse and socio-economically disadvantaged area of Amsterdam. The initiators were insufficiently able to influence patients with unhealthy lifestyles to change their behaviour through mainstream information and education. Patients with a combination of physical and mental health complaints represented a significant proportion of the total demand for care. Together with other professionals who worked in the health care centre, they developed an intervention consisting of group meetings focusing on physical activity, social support and enjoyment, combined with individual sessions to improve participants’ functioning. The group was coached by physical therapists and met weekly for three to four months. Participants were referred to the intervention by general practitioners.

Evaluations revealed that after the intervention, participants felt healthier and were able to manage their health conditions better. Participants increased their physical activity, had more social contacts in the neighbourhood, kept close contact with at least one participant from the group, and felt more self-confident. Moreover, the number of consultations with the GP decreased during the intervention [16,17,18]

In 2011, the intervention was further developed, focusing more specifically on the psychological aspects of functioning, because participants in the intervention often experienced mental health conditions. To further develop the intervention for this specific target group, a multidisciplinary, expert team was formed consisting of mental health care professionals, physical therapists and general practitioners. The team also consulted external business advisors for advice on the organisational aspects of implementing the intervention. They met weekly from December 2010 till December 2011. The experiences and expertise of participants and professionals of the first intervention informed the development process. The team focused on identifying core elements of the intervention and designing a format consisting of a combination of individual and group sessions.

## Theoretical frameworks

The core approach of the intervention BigMove is to enable participants to describe their functioning from their own perspective, to enable them to set their own goals regarding their functioning, and foster behaviour change both in individual and group sessions. Two theoretical frameworks, the Capability Approach (CA) and Self Determination Theory (SDT), provide the conceptual basis of the intervention. The essential elements of the intervention are grounded in the CA and the SDT. The frameworks are described below.

### Capability Approach

The Capability Approach (CA) was first developed by Sen [19] and further developed in collaboration with Nussbaum [20]. The CA is a broad, normative framework that concentrates on people’s real freedom to do the things they have reason to value and the quality of life that can be achieved when choosing between options available to them [20]. According to Sen, a socially just society must aim to strengthen people’s capabilities and provide freedom of choice to shape a dignified and meaningful life which will ultimately foster improvement in quality of life. The theory’s two core concepts are *functionings* and *capabilities*. Functionings are the achievements in being and doing and capabilities are the real freedoms or opportunities to do and be what you have reason to value [21]. The CA also emphasizes people’s ability to convert *resources* (means people have or public goods they can access) into capabilities and functionings. These are referred to as *conversion factors*, which can be internal (i.e. physical conditions, sex, skills) or external (i.e. social and environmental). Conversion factors are not fixed but are influenced by policies and personal choices [22,23]. Agency is an important aspect of the CA. According to Sen, an agent is someone who “acts and brings about change and whose achievements can be judged in terms of his own values and objectives” [21, p. 64]. Agency, therefore, refers to the ability of a person to act on what they value and have reason to value, and agency achievement refers to how successful a person is in achieving their goals [24]. Building on the work of Sen and Nussbaum, Mitra (2018) developed the Human Development Model to make explicit the relationship between health conditions, impairments, and wellbeing, as well as the social determinants influencing them. Health conditions in the model are viewed as conditions influenced by resources, structural and personal factors. Capabilities and functionings are used in the model as metrics for wellbeing. Wellbeing is considered as a multidimensional concept in which individual choices and values are central aspects [25].

### Self-Determination Theory

The Self-determination Theory (SDT), developed by Deci and Ryan, is a theoretical framework to explain human motivation. The theory focuses on psychological processes that promote optimal functioning and health [26].

SDT identifies three basic needs that should be fulfilled to promote behaviour change: autonomy, competence, and relatedness. Autonomy refers to the capacity to determine one’s own behaviour, from personal aspirations and values. Competence refers to the feeling of being effective and having the possibility to use capacities. Relatedness refers to feelings of belonging, safety, and security. Together these needs are the basis of ongoing psychological growth, integrity, and well-being [27]. Relatedness maintains or enhances intrinsic motivation and promotes or strengthens “aspirations or life goals that ongoingly provide satisfaction of the basic needs” [26 p.263]. Increased intrinsic motivation is associated with more adaptive behavioural and better health outcomes [26,28]. However, when these needs are not satisfactorily met, there is a negative impact on mental health functioning and performance [26].

## Essential elements of intervention BigMove

The intervention BigMove consists of basic elements that are combined to promote behaviour change, and which are described in detail below. The intervention is related to the CA as it aims to improve functioning and capabilities, in which individual choice and values are central aspects. The elements of the intervention are designed to enhance participants’ articulation of their personal choices and values, which also aligns with the basic need for autonomy as defined by SDT and is reflected in the setting of goals based on one’s own aspirations and values.

The elements also foster the two other basic needs defined by SDT: competences and the sense of relatedness of participants. Competences are increased through physical activity, the sharing of knowledge in groups sessions and in individual cognitive behavioural therapy sessions. Participants’ ‘sense of relatedness’ is strengthened by the social support of the group. Ultimately, the combination of all essential elements in the intervention improves functioning and capabilities and enables participants to fulfil their basic needs leading to “the realisation of the best within us by focusing on what matters most” [29].

### The International Classification of Functioning, Disability and Health (ICF) as a basis for motivational interviewing and goal setting

In line with the CA and the SDT, participants in the intervention are invited to set their own goals regarding their functioning, using the ICF as a basis. The ICF is both a theoretical framework and a classification for organising and documenting functioning and disability. Within the framework, the classification consists of more than 1500 categories over three domains (functions, activities and participation) and two contextual domains (external and personal factors) influencing functioning. The use of the ICF can reveal the impact of health conditions on people’s daily functioning and how people manage their health status. It provides a common, international language to measure and compare health information on people’s functioning from a broad biopsychosocial perspective [30].

It is important to note that functioning is defined differently in the two frameworks. In the ICF, functioning (written in singular) captures all domains of life (body functions and structures, activities and participation) and take into account contextual factors influencing functioning, whereas in the CA functionings (written in plural) refer to achievements in being and doing that people have reason to value [31]. In the intervention, functioning (written in singular, as it primarily refers to the ICF) is used in both ways, as participants describe their functioning in ICF terms and express on how they value their functioning by assigning subjective labels to ICF categories, in line with the CA.

Professionals involved in the intervention are trained in motivational interviewing to support participants to describe how they perceive their functioning across all domains. Motivational interviewing was first described by Miller [32]. As a tool for health coaching, motivational interviewing is effective in chronic care management and leads to increased self-efficacy, patient activation and perceived health status [33]. Markland et al. state that motivational interviewing provides social-environmental facilitating factors as described in the SDT and that “motivational interviewing impacts on perceptions of support for autonomy, competence, and relatedness; actual satisfaction of these needs; autonomous motivation for change; and subsequently on behaviour change and maintenance” [34, p. 826].

Professionals also support the participants to set their goals related to their functioning. Setting and achieving personal goals has a strong motivational effect. It increases the perception of competence and confidence that future goals can be achieved [35]. People who participate in determining the strategy to achieve goals perform better and have higher scores on self-efficacy than those who do not [36]. Internalisation of goals is also recognised as an important condition for actually achieving them [37].

### Cognitive behavioural therapy

In the intervention, the therapist aims to provide insights into the participant’s thought and behaviour patterns and guide participants in creating new habits. These insights are revealed through reflection on how the participant’s thoughts and behaviour are related to their actions and experiences in life and specifically in the group sessions. Cognitive behavioural therapy (CBT) is a widely used and studied form of psychotherapy. It combines cognitive, behavioural and emotion-focused techniques. A review of meta-analyses found that the evidence-base for the use of CBT to manage a wide variety of psychological symptoms is very robust [38]. In CBT, patients formulate hypotheses based on “their beliefs (theories) about the world, themselves, and their future” [39, p. 200]. CBT focuses on changing unhelpful thoughts and beliefs to “increase perceived ability of coping, reduce perceptions of personal vulnerability, and reduce emotional distress” [39, p. 200]. An active coping strategy positively influences mental health and will help to manage depressive symptoms [40]. If people can develop successful coping strategies, mental health is improved, depressive symptoms are easier to manage, and limitations have less impact on the quality of life [40,41]. Therapists who focus on the autonomous motivation for change and use CBT techniques facilitate greater and more sustainable change [42]. Professionals applying the intervention are trained to explore participants own motivation for change.

### Enjoyment and support of the group

The intervention’s group activities include elements of ‘play’ in order to foster joy. One of the basic capabilities, according to Nussbaum, is being able to laugh, play and enjoy recreational activities [43]. Enjoyment is also an essential motivator for a lasting lifestyle change and associated positive health effects [44,45].

Social support from the group is another important element of the intervention, as group work can be enjoyable, improve motivation and strengthen perseverance to complete the intervention [46,47]. Low motivation is one of the main reasons people with mental health conditions do not take up and adhere to physical activity. Social support positively influences emotions, wellbeing and coping with stress [48]. Also, inadequate social and emotional support has a detrimental influence on all health-related quality of life domains [49].

### Physical activity to foster physical and mental well-being

Physical activity is a central element of the group sessions in the intervention. It aims to improve physical and mental functioning and to stimulate social interaction. Research indicates that patients with chronic conditions feel and function better with greater levels of exercise [50] and physical activity improves self-rated health and quality of life [51,52]. Physical activity interventions that aim to improve mental health conditions also positively impact the symptoms of mental health conditions [52]. Furthermore, physical activity can be an intrinsic or extrinsic motivator for behaviour change and therefore in itself can become a motivational element of an intervention [53]. Physical activity as part of the intervention improves functioning and leads to increased motivation, as described in the SDT, resulting in better health outcomes.

### Complex dynamic coaching

Professionals are trained to foster the process of strengthening people’s functioning and capabilities. As the intervention is dynamic and considers the diversity and complexity of people’s lives, professionals are trained to address this dynamic complexity throughout the intervention. Therefore, the coaching of professionals is referred to as complex dynamic coaching. Professionals applying the intervention are taught how to create new experiences and interactions in the group sessions through combining the essential elements that in turn lead to an expansion of the behavioural repertoire of the participants.

The coaching focuses on enhancing knowledge, competences and attitudes; providing new challenges; receiving and acting on feedback; developing social support within the group; physical activities; and experiencing enjoyment. The participants set group goals to enhance the social cohesion of the group. The coaching resembles the theory of nonlinear pedagogy (NLP). Essential elements of NLP are “a variety of modified games, the freedom to choose, an emphasis on exploration and problem-solving” [54, p. 29]. The approach refrains from judgment or give instructions on how to act. Research indicates that NLP facilitates the improvement of the basic needs of SDT; perceived competence, autonomy, and relatedness. Improvement of the basic needs of SDT might in turn lead to improvement of intrinsic motivation and enjoyment during practice [53]. The training for coaches was also developed by the multidisciplinary team that developed the intervention.

## Implementation of the care practice

### Target group

Potential participants of the intervention are patients with a combination of physical and mental health conditions. People with combined physical and mental health conditions typically visit their general practitioner frequently. They often exhibit unhealthy lifestyle behaviours. Usually, various other health care professionals and social care workers are involved in their care.

### Inclusion and exclusion criteria

Inclusion and exclusion criteria for referral to the intervention are:

**Table:** 


INCLUSION	EXCLUSION

– 18 years and older – Presence of a mental health condition (such as mood, anxiety, behavioural or somatisation disorders) in combination with somatic and social conditions – Conditions have a profound impact on functioning in multiple areas of life. – Physically capable of participating in the intervention.	– Behaviour that disrupts group processes: threats, aggression, impulse control disorder, severe contact disorder etc. – Psychotic state (e.g. delusions, hallucinations) – Criminal behaviour related to the psychiatric disorder. – Physically unable to participate in the intervention.


People without a permanent residence or insurance are not specifically excluded from the intervention, however they are seldom referred.

### Setting

The intervention is organized in the community or neighbourhood of the participants, thus offering the possibility to engage in local activities and the continuation of support of the group once the intervention is completed. Over 25 locations in the Netherlands have implemented the intervention in neighbourhood settings with local professionals. The location of the group intervention varies, most commonly a gym in a physical therapy centre, a school sports hall, or community centre. Groups also sometimes use the local outdoor settings.

### Training for professionals

The intervention staff involved at each location consists of two mental healthcare professionals (healthcare psychologist and a clinical psychologist/psychiatrist) and two BigMove professionals, mostly physical therapists. The staff is trained during a three-day training. The training aims to change perspectives on treating physical and mental health conditions. The training develops individual and groups coaching competences, knowledge about the structure and implementation of the intervention, and the specific tasks of each professional in the interdisciplinary care team. During the intervention, all professionals can consult the coordination team for support. As a follow-up to the training, there are regular opportunities for continuous education and on-the-job coaching. Before the start of the intervention the general practitioners in the local community are informed about which patients are suitable for referral to the intervention.

### Materials

Several materials were developed to support the implementation of the intervention.

– An e-health application based on the ICF for registration of functioning and setting of goals; the app is installed on an iPad and used by professionals together with participants.
– Training materials for professionals.
– A leaflet with information for participants and referring health care professionals.

The materials are drawn up in Dutch and are accessible to all professionals who implement the intervention.

## Structure of the intervention

The intervention consists of individual and group components with a total duration of approximately six months. Each group is composed a priori and consists of 12 to 15 participants. Two BigMove professionals lead the group sessions. Individual sessions are scheduled with one BigMove professional, each participant is assigned to a BigMove professional before the start of the intervention.

**Table:** 


INDIVIDUAL SESSIONS	GROUP SESSIONS

**Intake** by the healthcare psychologist and the clinical psychologist or psychiatrist for the determination of a psychiatric diagnosis and the preparation of the treatment plan together with the patient (shared decision-making). Feedback to the referee about inclusion or exclusion criteria of the intervention **First session with the BigMove professional.** Assessment of functioning and setting of goals. **Three to ten individual sessions with healthcare psychologist for cognitive** behaviour therapy **Two to five individual sessions with the BigMove professional** to discuss individual progress. Previous goals are assessed and adjusted where necessary. **Interdisciplinary consultation:** Two to four consultations during the intervention between the participant, healthcare psychologist, the clinical psychologist/psychiatrist and the BigMove professional. **Final evaluation with the healthcare psychologist.** Evaluation with the participant. In addition, the patient is assessed by the clinical psychologist or psychiatrist.	**Fifteen group meetings** of one-and-a-half hours per week. The BigMove professionals use Complex Dynamic Coaching (CDC) in the group sessions. The content of the group sessions is not predefined but follows the group preferences and process of the group. The group can request guest sessions, for example, with a nutritionist or a yoga teacher. The process and sequence of the sessions are the same, but the form can be different every time to provide new challenges. – *Group session 1: start the group process, define group goals and discuss group rules* – *Group sessions 2-5: focus on awareness, motivation and trust*. – *Group session 6: intermediate evaluation and possible adjustment of group goals*. – *Group session 7-11: focus on participation and social support, building relationships, inviting a participant’s close relative or friend to a group meeting, and helping others*. – *Group session 12: intermediate evaluation and possible adjustment of group goals*. – *Group session 13-14: focus on motivation, perseverance, and continuity of activities; trying out various activities in the neighbourhood and committing to one of these activities* – *Group session 15: end meeting and evaluation*


## Patient case

The following patient case illustrates the experience of participating in the intervention.

“I’ve been suffering from an anxiety disorder for quite some time, and I’ve had several therapies. At the beginning of 2014, I had a difficult period again. When I went to the doctor with my complaints, he advised me to go to BigMove. He had good experiences with this intervention. It seemed like a nice trajectory because I could get in touch with my feelings in a different way. I already had had a lot of conversational therapies in a one-on-one setting. BigMove looked very different in terms of content. I love sports and exercise. That’s why it appealed to me.

I liked the group, the group dynamics, the ‘we’ feeling, a feeling of belonging. That worked well for me. The conversations with the BigMove healthcare psychologist were also useful. They were often about the group meetings and the things I experienced in them. They brought me closer to myself.

I learned a lot about an exercise in which I had to cross my boundaries. It turned out that I was able to do that really well. It strengthened my self-confidence. I discovered that I am physically and sensitively balanced. My peers confirmed me in this. Their reaction did a lot to me; I had always thought the opposite about myself.

Being in motion has released good signals in my head. If your psyche doesn’t function properly anymore, you can feel it in your body. With me it was and still is high breathing in my throat, sore shoulders and headaches.’

I’ve learned that these signals tell me I’m crossing my boundaries. I have to take good care of myself. For me this means, for example, meditating, no alcohol and keep up running, even though I don’t feel like it!

Before BigMove, I especially saw myself as someone with an anxiety disorder. Through BigMove, I’ve experienced that I’m so much more than that. I know now that I have a strong capacity for empathy and that I’m a go-getter. I’m optimistic and I want to make something of my life. I take initiative and I’m an inspiration to others. That’s why I started studying Lifestyle Coaching. I want to help people from my own experience.

I got a helicopter view at BigMove. Ah, this is happening inside me. That gave me insight!”

## Discussion

People with physical and mental health conditions need and deserve better care. The current single, disease-oriented approach often fails to be effective or efficient and is increasingly recognised as inappropriate for the management of (multiple) chronic conditions. The intervention BigMove, as a group -based intervention for people with physical and mental health conditions, shows how integrated care can be organised to strengthen people’s functioning and capabilities. It was initiated in 2011 and applied in more than 25 locations in the Netherlands.

The intervention has a strong theoretical foundation. Two theoretical frameworks, the CA and SDT, formed the bases of the development process and design of the intervention and are reflected in the essential elements of the intervention. The CA addresses the importance of aligning care with the personal values and aspirations of participants. SDT expresses the importance of fostering motivation through the three basic needs: autonomy, competence, and relatedness. These needs are reflected in all components of the intervention.

The intervention focuses on strengthening participants capabilities and intrinsic motivation for change, which is in line with findings of a recent review on conceptualisations of person-centred care that concludes that “person-centred healthcare must value the social network of each patient, promote quality of life and personal goals, not only health status improvement” [55, p. 2]. A recent study on complex interventions recommends that elements of biological, psychological, social, cognitive behavioural, and environmental support should be considered when constructing such interventions [56]. These domains are all reflected in the intervention BigMove.

In the intervention, multiple professionals collaborate and align the sessions according to goals set both by individuals and by the group. Although all elements are considered essential to positively influence functioning, support of the group is perhaps the most fundamental aspect that helps participants complete the intervention. Indeed, many participants continue to support each other after the intervention. The setting of the intervention is also important. The intervention is organized in the local environment of the participants, which offers the possibility to engage in local activities and the continuation of group support once the intervention is completed. The collaboration of multiple professionals (mental health care professionals, physical therapists, and general practitioners) provides a basis to improve the coordination of care and long-term health outcomes. In 2015, the intervention was included in the national mental health standard of health promotion for people with severe mental health conditions. The standard was developed by a working group with representatives of patients, healthcare professionals and researchers, in which they jointly identify good care examples [57].

The complexity of the intervention can influence implementation. All professionals involved need to be well informed and trained to carry out the intervention. The design of the intervention requires that professionals adopt a new approach in their delivery of care, both in their attitude and in the application of all elements combined. In their attitude, they should focus on stimulating functioning and capabilities. Moreover, they should understand the rationale and use of each element in the intervention. This requires both the ability to execute each element and the ability to combine them. The training requires time investment of the professionals who will execute the intervention. Also, general practitioners need to be informed which patients can benefit from and meet the inclusion criteria of the intervention.

To date, there is still limited evidence about the effectiveness of interventions for people with both physical and mental health conditions. Research has been mostly limited to interventions for physical comorbidities. Group-based interventions that aim to improve overall functioning and well-being, do show positive results on mental health functioning and well-being [58,59]. However, comparison between these interventions and the intervention BigMove is complicated by the diversity of their target groups or the aim of specific elements such as social participation [60]. We are not aware of other group-based interventions that integrate care to specifically improve both physical and mental health. A review published in 2010 already pointed out the increasing importance of developing interventions for long-term conditions that consistently provide benefits for both physical and mental health and the authors conclude that integrated care with a biopsychosocial foundation is difficult to achieve [61].

Since BigMove is holistic and involves a multidisciplinary approach, combining individual and group sessions, it does not fit in the current funding of mental health care. To enable person-centred care, it is not only crucial to reform the interaction between professionals and patients but also to re-structure the organisation of care to enable continuity of care to improve “the experience the patient has in interacting with the wider healthcare system” [55]. Furthermore, the duration of the programme is relatively long. Yet, a review has demonstrated that longer interventions that combine CBT with exercise tend to have a greater impact on reducing depression and anxiety symptoms [62].

Finally, evaluating a complex intervention that consists of many elements and draws on diverse theoretical frameworks is a challenge. This is further complicated as “complex interventions continually adapt to local contexts, making stability and fidelity unlikely” [63, p. 241]. Various methods can be used to evaluate a complex intervention and the choice of method depends on what aspects (such as the effect of the intervention, implementation, mechanisms of impact, the effect of contextual factors) are evaluated [64]. Possible methods to evaluate complex intervention include mixed-methods design and multiple single-subject design [65,66]. According to the 2019 updated guide on developing and evaluating complex interventions, evaluations should include the assessment of effectiveness, understanding of the change process and assessing cost-effectiveness [67]. Evaluations should focus on an improvement of people’s functioning and capabilities, rather than solely on a decrease of symptoms of a disease. Using the ICF in conjunction with the CA to reflect people’s functioning, as well as how they perceive and value their own functioning and capabilities, could be a starting point of such evaluations to improve our understanding of quality of life for people living with chronic health conditions [68]. As proposed by Madden et al [2015], an Integrative Measurement of Functioning (IMF) based on ICF could be used to provide a common, positive, language and measurement of functioning which might also support person-centred care [69].

Lessons learned:

– To meet the complex needs of people with both physical and mental health conditions a biopsychosocial approach is required.
– The intervention BigMove is based on well-developed theoretical frameworks and consists of multiple essential elements, aimed at improving functioning, capabilities and quality of life for people with physical and mental health conditions.
– The combination of individual and group sessions, focusing on personal goal setting, group support and physical exercise, can foster behaviour change.
– Future research is needed to improve the interventions’ implementation and evaluate its effectiveness.

## Conclusion

We conclude that for people with physical and mental health conditions, the intervention BigMove is a promising approach, as it addresses the aspirations people have regarding their functioning and focuses on individual goal setting. The theoretical basis of the CA and SDT are reflected in the essential elements of the intervention, resulting in a coherent approach, aimed at improving functioning, capabilities and quality of life. A next step is to further implement the intervention and evaluate its effectiveness.
